# Isolation, cultivation and identification of human lung adenocarcinoma stem cells

**DOI:** 10.3892/ol.2014.2639

**Published:** 2014-10-24

**Authors:** DE-GENG ZHANG, AI-GUI JIANG, HUI-YU LU, LI-XIN ZHANG, XIAO-YAN GAO

**Affiliations:** 1 Department of Oncology, Taizhou People’s Hospital Affiliated to Medical College of Nantong University, Taizhou, Jiangsu 225300, P.R. China; 2 Department of Respiratory Medicine, Taizhou People’s Hospital Affiliated to Medical College of Nantong University, Taizhou, Jiangsu 225300, P.R. China; 3 Institute of Clinical Medicine, Taizhou People’s Hospital Affiliated to Medical College of Nantong University, Taizhou, Jiangsu 225300, P.R. China

**Keywords:** lung cancer stem cell, lung cancer, cell separation, cluster of differentiation 133

## Abstract

Recently, an increasing number of studies have demonstrated that lung cancer is a stem cell disease. However, ideal cell surface markers for isolating stem cells in lung cancer are yet to be identified. In the present study, a cell population with a cluster of differentiation (CD)133^+^ phenotype was successfully isolated from a single cell suspension of lung adenocarcinoma tissue using magnetic-activated cell sorting (MACS) and enriched in a serum-free culture. In comparison to CD133^−^ cells, the CD133^+^ cells exhibited an enhanced capacity for self-renewal and differentiation, and a greater potential for *in vivo* tumor formation, in non-obese diabetic/severe combined immunodeficient (NOD/SCID) mice. Tumors could be induced in NOD/SCID mice by the transplantation of 10^2^ stem-like cells per mouse. The results of the present study demonstrated that CD133 may serve as a specific cell surface marker for lung adenocarcinoma stem cells, and that MACS combined with serum-free culture is an effective method for isolating and enriching lung cancer stem cells.

## Introduction

Non-small cell lung cancer (NSCLC) is one of the leading causes of cancer-related mortality worldwide, despite considerable progress in surgery, chemotherapy, radiotherapy and biological targeted therapy (1). In the previous decade, NSCLC research has demonstrated that these therapies predominantly function to improve the patient’s quality of life and that the overall five-year survival rate for such tumors is <15% (2). Previous studies indicate that various solid tumors, such as brain gliomas (4,5), breast (6), prostate (7), colon (8) and liver cancers (9), contain a small population of cancer stem cells (CSCs) that are responsible for tumor maintenance and dissemination. CSCs exhibit unlimited proliferative potential, the ability to self-renew, an elevated capacity to induce malignancy, and may be associated with the initiation and progression of malignancies, chemotherapy and radiotherapy resistance, as well as tumor recurrence and metastasis (10–14). Since the characteristics of these tumor cells are similar to those of classic stem cells, they have been termed CSCs. It is proposed that therapies specifically targeting the stem cell signaling pathways utilized by CSCs may be beneficial in combating specific types of cancer (15–16).

Initial research into lung CSCs (LCSCs) has been undertaken in recent years. For example, Kim *et al* (17) identified a group of cells at the bronchioalveolar duct junction carrying Clara and alveolar markers, which commenced division following naphthalene administration-induced damage. This cluster of stem cell antigen-1 (Sca-1)^+^/cluster of differentiation (CD)34^+^ cells was enriched by fluorescence-activated cell-sorting, and demonstrated an enhanced capacity for self-renewal and differentiation *in vitro*. Activation of the oncogenic protein K-ras boosted the proliferation of the double-positive cells and accelerated tumorigenicity. Thus, the Sca-1^+^/CD34^+^ cells were termed bronchioalveolar stem cells (BASC) and may be the origin of adenocarcinomas. Furthermore, Ho *et al* (18) used flow cytometry and the Hoechst 33342 dye efflux assay to isolate and characterize side population (SP) cells from six human lung cancer cell lines (H460, H23, HTB-58, A549, H441 and H2170) and sixteen clinical lung cancer samples. The study performed xenograft experiments to determine that SP cells were enriched in tumor-initiating capability compared with non-SP cells, as well as Matrigel invasion assays to demonstrate that SP cells exhibit a higher potential for invasiveness. In addition, SP cells displayed elevated expression levels of ATP-binding cassette superfamily G member 2 (ABCG2), as well as other ABC transporters, and exhibited resistance to multiple chemotherapeutic agents. These findings indicate that SP cells are an enriched source of lung tumor-initiating cells with stem cell properties. Recent study has demonstrated that SCLC and NSCLC contain cells that express the glycoprotein prominin-1 (CD133), a cancer stem cell marker, which is essential for tumor cell propagation and metastasis (19). The proliferative capacity of CD133^+^ cells is yet to be determined; however, it is hypothesized that these cells serve as a reservoir for generating further cancer cells that are capable of tumorigenesis, leading to metastasis (19).

In the present study, a population with a CD133^+^ phenotype cells from a single cell suspension of lung adenocarcinoma tissues was isolated using magnetic activated cell sorting (MACS) technology and enriched in a serum-free culture. Furthermore, the self-renewal, differentiation and tumorigenicity of CD133^+^ cells in NOD/SCID mice were investigated.

## Materials and methods

### Cell culture and lung cancer single cell suspension preparation

Eight fresh lung cancer specimens were obtained (Table I) from patients who underwent surgery at the Department of Throacic Surgery, Taizhou People’s Hospital (Taizhou, China) between February and April 2013. The specimens were cut into 0.5-mm sections following the removal of visible blood vessels and necrotic tissue. The tissue specimens were washed numerous times with D-Hank’s solution (Beijing Huamaike Biotechnology Co., Ltd., Beijing, China) and left overnight in Dulbecco’s modified Eagle’s medium with Ham’s nutrient mixture F-12 (DMEM-F12) supplemented with high doses of penicillin/streptomycin and amphotericin B to avoid contamination. The specimens were enzymatically digested in 50 ml BD Falcon™(Becton-Dickinson, Franklin Lakes, NJ, USA) supplemented with collagenase IV (final concentration, 0.1%; Nanjing Sunshine Biotechnology Co., Ltd., Nanjing, China) and hyaluronidase (final concentration, 0.1%; Nanjing Sunshine Biotechnology Co., Ltd.) for 1 h under 5% CO_2_ at 37°C. The remaining cell debris was removed by passing the cells through a 70μm-diameter disposable cell mesh filter and centrifuging for 15 min at a speed of 400 × g. Finally, the primary human lung cancer cells were cultured in RPMI-1640 supplemented with small airway growth medium (SAGM) SingleQuots™ kit (Lonza, Basel, Switzerland) medium with penicillin/streptomycin, and features of the growth pattern were observed. Flow cytometry was performed three times using an EPICS^®^ XL™ flow cytometer (Beckman Coulter, Brea, CA, USA) to quantify the expression of CD133 (Miltenyi Biotec, Inc., Auburn, CA, USA) on the surface of primary human lung cancer cells. The present study was approved by the Ethics Committee of Taizhou People’s Hospital (Taizhou, China) and was performed according to the Declaration of Helsinki. Written informed consent was obtained from the family of each patient.

### CD133 cell sorting using immunomagnetic beads

A single cell suspension of ~1×10^7^ lung cancer cells was used for cell sorting. Cells were incubated with CD133/l rabbit anti-human polyclonal immunomagnetic beads (Miltenyi Biotec, Inc.) for 30 min at 4°C. For magnetic separation, a MACS column (Miltenyi Biotec, Inc.) was used to retain the positive cells linked with the beads. The CD133^+^ cells obtained from the column were centrifuged and resuspended in serum-free DMEM-F12 medium containing 50 μg/ml insulin, 100 μg/ml apo-transferrin, 10 μg/ml putrescine, 0.03 mM sodium selenite (all from Sigma-Aldrich, St. Louis, MO, USA), 2 mM progesterone (Pure Chemistry Scientific Inc., Sugarland, TX, USA), 0.6% glucose (LGM Pharma, Nashville, TN, USA), 5 mM HEPES (Nanjing Search Biotech Co., Ltd., Nanjing, China), 0.1% sodium bicarbonate (Nanjing Search Biotech Co., Ltd.), 0.4% bovine serum albumin (BSA; Wuhan Boster Bio-Engineering Co., Ltd., Wuhan, China), glutamine (Amresco LLC, Solon, OH, USA) and antibiotics (Gibco-BRL, Carlsbad, CA, USA) supplemented with 20 μg/ml epidermal growth factor (EGF; PeproTech EC Ltd., London, UK) and 10 μg/ml basic fibroblast growth factor (bFGF; PeproTech EC Ltd.). The purity of the CD133^+^ and CD133^−^ cells was evaluated using standard flow cytometric analysis. The CD133^+^ and CD133^−^ cells were harvested, and sphere formation, tumorigenicity and differentiation activity were determined.

### Cell surface marker analysis by flow cytometry

Cells (1×10^5^)were resuspended in 100 μl phosphate-buffered saline (PBS) supplemented with 0.5% BSA and 2 mM EDTA, and incubated with 10 μl polyclonal mouse anti-human CD133-PE conjugated antibody (1:100; Miltenyi Biotec, Inc.), monoclonal mouse anti-human cytokeratin (CK)8 (1:100) and mouse anti-human CK18 (1:100; Dako, Glostrup, Denmark) for 10 min at 4°C. Following washing with PBS, the cells were resuspended in a solution of PBS and 2 μl 7-amino-actinomycin D (7-AAD), and analyzed using a EPICS^®^ XL™ flow cytometer (Beckman Coulter).

### Immunofluorescence

Slides containing CD133^+^ tumor spheres and CD133^−^ cells were collected and immersed in PBS for 5 min, permeabilized in 0.1% Triton X-100 (Bebco Industries Inc., La Marque, TX, USA) for 10 min and washed with PBS 3 times for 5 min. Following blocking with 5% BSA at 37°C for 30 min, the slides were incubated overnight with rabbit polyclonal anti-human CD133 (dilution, 1:300; Abcam, Cambridge, UK) at a temperature of 4°C. Subsequently, the cells were incubated with Cy3-conjugated monoclonal goat anti-rabbit IgG secondary antibody (1:2,000; Wuhan Boster Bio-Engineering Co., Ltd.) diluted with 1% BSA at 37°C for 1 h. Finally, the cell nuclei were stained with DAPI (dilution, 1:200). Images were captured and visualized using fluorescence microscopy (AF6000; Leica, Mannheim, Germany).

### Sphere-forming assay

CD133^+^ tumor spheres and CD133^−^ cells were dissociated into single-cell suspensions, and transferred to 96-well plates. The cells were cultured in serum-free DMEM-F12 medium containing 50 μg/ml insulin, 100 μg/ml apo-transferrin, 10 μg/ml putrescine, 0.03 mM sodium selenite (all from Sigma-Aldrich), 2 μM progesterone (Pure Chemistry Scientific Inc.), 0.6% glucose (LGM Pharma), 5 mM HEPES (Nanjing Search Biotech Co., Ltd.), 0.1% sodium bicarbonate (Nanjing Search Biotech Co., Ltd.), 0.4% BSA (Wuhan Boster Bio-Engineering Co., Ltd.), glutamine (Amresco LLC) and antibiotics, supplemented with 20 ng/ml EGF and 10 ng/ml bFGF. Wells containing greater than one cell or no cells were marked and dismissed from statistical analysis. The cells were cultured in 5% CO_2_ at 37°C for 2–3 weeks, with the medium replaced or supplemented with fresh growth factors twice a week. Wells that contained spheres were counted using inverted phase contrast microscopy (DMI 6000B; Leica) and the percentage of cells exhibiting sphere-forming capacity was calculated.

### Differentiation

CD133^+^ tumor spheres and primary lung cancer cells were cultured in 24-well plates. To allow cell attachment and differentiation, the stem cell medium was replaced with RPMI-1640 supplemented with SAGM SingleQuots kit medium with penicillin/streptomycin and 10% fetal bovine serum (FBS; GE Healthcare Life Sciences, Logan, UT, USA). The acquisition of differentiation markers and the loss of stem cell markers was evaluated using flow cytometry before and after cell attachment, as described above. The experiment was repeated three times and the mean values were calculated.

### Chemotherapy resistance studies

Cells (1×10^3^) obtained from CD133^+^ tumor spheres and CD133^−^ cell dissociation were plated in 96-well flat-bottomed plates. The chemotherapeutic agents gemcitabine and cisplatin (Jiangsu Hansoh Pharmaceutical Co., Ltd., Lianyungang, China) were added at final concentrations of 250 mM and 5 mg/ml, respectively. Following six days of treatment, cell viability was evaluated using an MTT and Trypan blue (Nanjing Search Biotech Co., Ltd.) exclusion assay. Data are expressed as the mean of three independent experiments performed with the two experimental procedures.

### Tumorigenicity in NOD/SCID mice

CD133^−^ cells and CD133^+^ tumor spheres were mechanically dissociated to obtain single cell suspensions and diluted in growth factor-containing medium prior to subcutaneous injection. Serial dilutions (10^2^, 10^3^, 10^4^ and 10^5^ cells) of the cells were subcutaneously injected into the abdominal wall of 20 four-week-old NOD/SCID mice (five mice per group; Beijing Vital River Laboratory Animal Technology Co., Ltd., Beijing, China). Tumor size was measured using calipers and tumor volume was calculated using the equation: Tumor volume = (π × maximum length × maximum width × maximum height)/6. Immunohistochemistry (IHC), as well as hematoxylin and eosin staining (H&E), were performed to analyze the tumor histology and to compare mouse xenografts with patient tumors.

### IHC

Paraffin-embedded tissue blocks were cut into 4-μm sections and representative sections were analyzed immunohistochemically (EliVision™ Plus IHC kit; Wuhan Boster Biological Engineering Co., Ltd., Wuhan, China) for mouse anti-human polyclonal CK8 and CK18 (dilution, 1:200; Miltenyi Biotec, Inc.). Briefly, the sections were dewaxed in xylene and rehydrated in ethanol using graded concentrations of alcohol. Endogenous peroxidase activity was blocked by incubating the sections in 5% hydrogen peroxide and absolute methanol at room temperature for 10 min, and antigen retrieval was performed in a microwave oven for two cycles of 10 min each. Primary antibodies were applied for 1 h at room temperature, the sections were washed three times with 0.05 M Tris-buffered saline (TBS; pH 7.2) and 50 μl IgG/horseradish peroxidase secondary antibody (Wuhan Boster Biological Engineering Co., Ltd.) was added, followed by incubation for 30 min at room temperature. The sections were washed three times with TBS and the reaction products were visualized using a diaminobenzidine (DAB) kit (Wuhan Boster Biological Engineering Co., Ltd.). The sections were counterstained with H&E, dehydrated and evaluated under a light microscope (DM 3000; Leica).

### Statistical analysis

Statistical analysis was performed using SPSS software (version, 13.0; SPSS, Inc., Chicago, IL, USA). All experiments were performed a minimum of three times and representative results are presented as the mean values ± standard deviation. Statistical analysis was performed by one-way analysis of variance and comparisons among groups were achieved using independent sample t-tests. P<0.05 indicated a statistically significant difference.

## Results

### CD133^+^ cells in primary lung cancer cells

**Figure 1:**
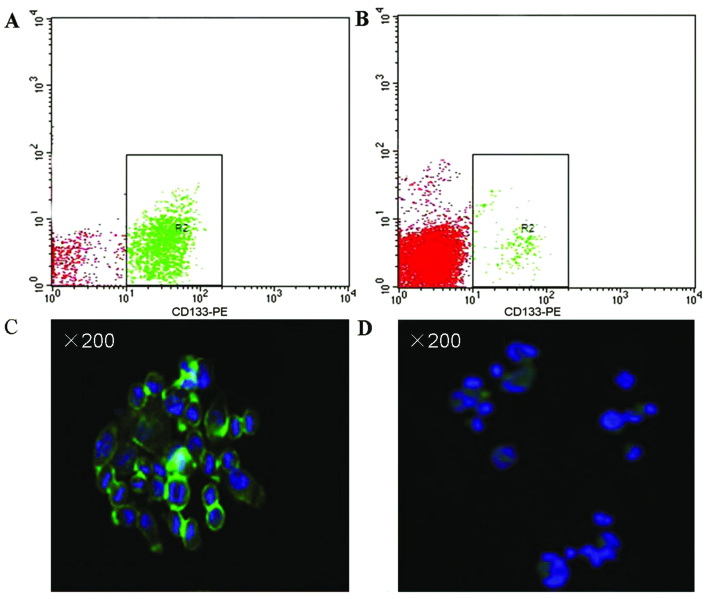
EPICS^®^ XL™ flow cytometry revealed (A) a high CD133^+^ expression rate in the CD133^+^-sorted population and (B) a low CD133^+^ expression rate in the single-cell suspensions of primary lung cancer cells (89.15±7.80% vs. 2.07±0.21%; P<0.01). Each experiment was repeated three times. Fluorescence microscopy of immunostaining assays (C) detected extensive expression of CD133 in the sphere samples; however, (D) lower levels of CD133 expression were detected in the primary lung cancer cells (staining, DAPI). Green staining presents positive cells. CD, cluster of differentiation.

CD133^+^ cells were detected in 4/8 primary human lung cancer samples using the EPICS XL flow cytometer (Beckman Coulter). The proportion of CD133^+^ cells was 1.9%, 2.1%, 1.3% and 0.8% in each respective case, and the pathological types of all four cases were lung adenocarcinoma (Table I). Immunomagnetic beads identified two cases of primary lung cancer cell suspension exhibiting a high percentage of CD133^+^ cells for cell sorting. Following immunomagnetic sorting with CD133 beads, CD133^+^ tumor cells were cultured in serum-free DMEM-F12 medium supplemented with 20 ng/ml EGF and 10 ng/ml bFGF, and analyzed using flow cytometry (EPICS XL flow cytometer; Beckman Coulter). Three measurements of CD133 expression in the CD133^+^-sorted population indicated a high mean CD133^+^ expression rate of 89.15±7.80% (Fig. 1A). However, the CD133^+^ expression rate in the single-cell suspensions of primary lung cancer was only 2.07±0.21% (Fig. 1B). Additionally, immunostaining assays demonstrated extensive expression of CD133 in the tumor sphere samples (Fig. 1C), while lower levels of CD133 expression were detected in the primary lung cancer cells (Fig. 1D).

### CD133^+^ cell enrichment in the serum-free cultures

**Figure 2:**
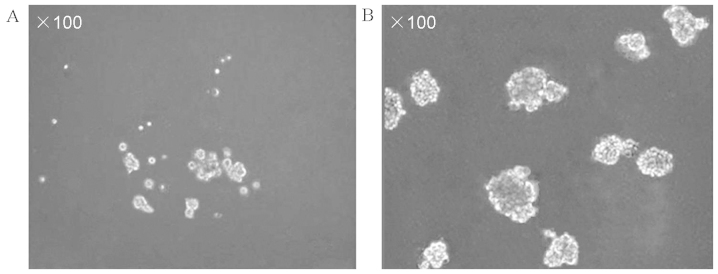
Phase contrast microscopy, demonstrating (A) CD133^+^ cells gradually formed sphere colonies of different sizes and irregular shapes following approximately one week of culturing and (B) CD133^+^ cells were exclusively formed of cellular clusters of tumor spheres following approximately one month of culturing in serum-free medium containing various growth factors. CD, cluster of differentiation.

According to the CSC theory, only a small number of cells in the tumor exhibit CSC characteristics. These stem-like cells are able to grow in serum-free medium and are innately resistant to chemotherapy, due to their ability to pump out toxic agents. The CD133^+^ cells obtained via immunomagnetic cell sorting were harvested and cultured in serum-free medium containing various growth factors. Following three days of culturing, numerous individual cells in the CD133^+^ suspension culture were observed to survive and proliferate. After approximately one week, these cells gradually formed sphere colonies of various sizes and irregular shapes (Fig. 2A). After approximately one month, these cell cultures were exclusively formed by cellular clusters resembling tumor spheres (Fig. 2B). However, in standard stem cell medium cultures, almost no clear sphere colonies were observed; the majority of CD133^−^ cells died within two weeks, and just a small number of CD133^−^ cells adhered to the wall and grew slowly.

### Sphere-forming assay

**Figure 3:**
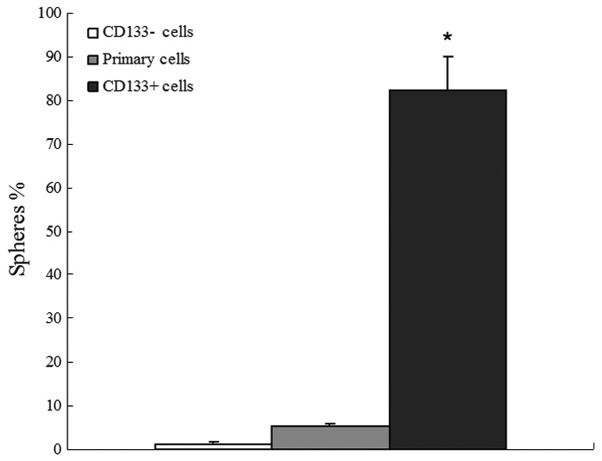
Sphere-forming assay revealed that CD133^+^ cells exhibited an enhanced capacity for self-renewal, compared with primary lung cancer and CD133^−^ cells (82.37±7.6 vs. 5.23±0.71 and 1.21±0.42%, respectively). ^*^P<0.01, compared with primary lung cancer and CD133^−^ cells. Each experiment was repeated three times. CD, cluster of differentiation.

The primary lung cancer cell suspension, CD133^−^ cells and CD133^+^ tumor spheres were examined for their ability to form new spheres following initial culturing as single cells. Subsequent to two weeks of culturing, 82.37±7.6% of single cell wells derived from CD133^+^ tumor spheres formed a new set of spheres; however, only 5.23±0.71% of wells with a single cell derived from the primary lung cancer cell suspension formed spheres. Additionally, almost no obvious sphere colonies derived from CD133^−^ cells were identified (Fig. 3).

### Differentiation of CD133^+^ cells

**Figure 4:**
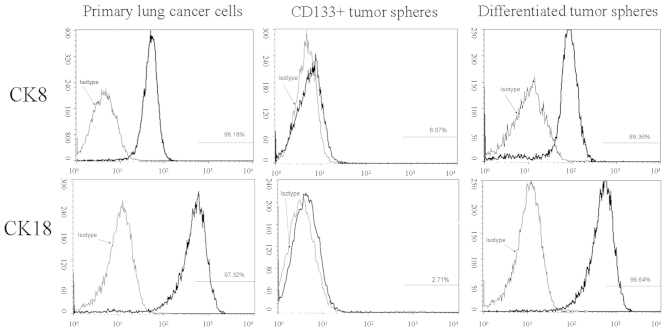
EPICS^®^ XL™ flow cytometry revealed that the expression of CK8 and CK18 in CD133^+^ cells increased from 6.07±0.32 to 89.36±9.08% (P<0.01) and 2.71±0.18 to 98.64±10.13% (P<0.01), respectively, following the cell adherence and tumor sphere differentiation in the culture system supplemented with 10% fetal bovine serum. Similarly, the CK8 and CK18 expression of primary lung cancer cells was 98.18±12.59 and 97.32±11.22%, respectively. Each experiment was repeated three times. CD, cluster of differentiation; CK, cytokeratin.

The expression of CK8 and CK18 in primary lung cancer cells, and before and after differentiation of CD133^+^ tumor spheres, was observed using flow cytometry. CK8 and CK18 expression increased from 6.07±0.32 to 89.36±9.08% (P<0.01) and 2.71±0.18 to 98.64±10.13% (P<0.01), respectively, following cell adherence and tumor sphere differentiation in the culture system supplemented with 10% FBS. Similarly, the CK8 and CK18 expression of primary lung cancer cells was 98.18±12.59 and 97.32±11.22%, respectively. This finding indicates that CD133^+^ cells have the potential to differentiate into various cell types (Fig. 4).

### CD133^+^ are cells resistant to conventional chemotherapy

**Figure 5:**
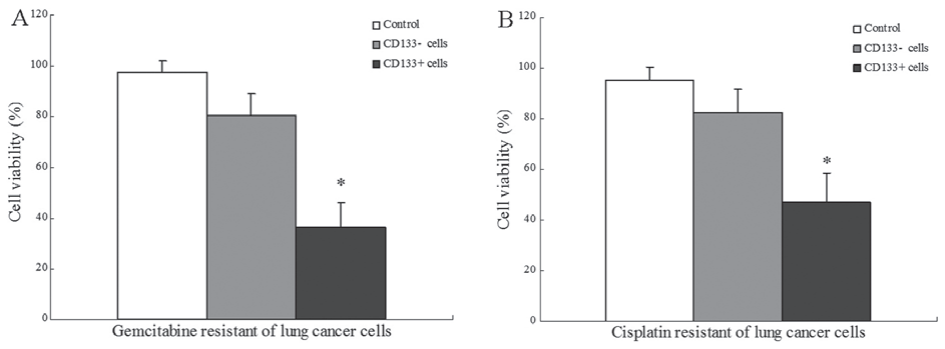
Chemotherapy resistance studies revealed that the resistance of CD133^+^ cells to (A) gemcitabine and (B) cisplatin was significantly higher than that of CD133^−^ cells *in vitro*. ^*^P<0.01, compared with CD133^+^ and control cells. Each experiment was repeated three times. CD, cluster of differentiation.

Gemcitabine and cisplatin were administered at doses comparable with the higher plasma levels obtained in treated CD133^+^ and CD133^−^ cells. The results demonstrated that the gemcitabine (Fig. 5A) and cisplatin (Fig. 5B) resistance of CD133^+^ cells was significantly stronger than that of CD133^−^ cells, *in vitro*.

### Tumorigenic potential of CD133^+^ tumor spheres in NOD/SCID mice

**Figure 6:**
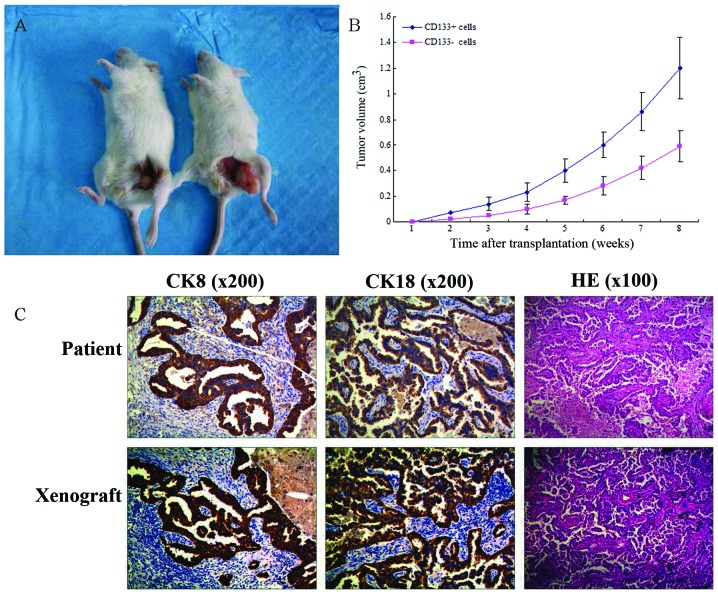
(A) Image of non-obese diabetic/severe combined immunodeficient mouse tumors. (B) Xenograft tumor volume induced by 10^5^ CD133^+^ cells was significantly greater compared with the xenograft tumor volume induced by 10^5^ CD133^−^ cells. (C) HE staining and immunohistochemical markers revealed that the xenograft and primary tumors exhibited similar histologies. CD, cluster of differentiation; CK, cytokeratin; HE, hematoxylin and eosin.

Accumulating evidence indicates that CSCs exhibit powerful tumorigenicity. To evaluate the hypothesis that CD133^+^ tumor spheres are more tumorigenic due to their enhanced stem-like properties, CD133^+^ tumor spheres and CD133^−^ cells were subcutaneously injected into NOD/SCID mice in a limiting dilution assay (10^2^, 10^3^, 10^4^ and 10^5^ cells; Fig 6A). The results demonstrated that CD133^+^ cells possess more powerful tumorigenicity compared with CD133^−^ cells, *in vitro* (Table II; Fig 6B). In order to further compare the size of the xenograft tumor of the two groups cells *in vitro*, the tumors induced by 10^5^ CD133^+^ and CD133^−^ cells were observed; the tumor volume induced by CD133^+^ cells was significantly greater than that induced by CD133^−^ cells. Additionally, H&E staining and immunohistochemical markers indicated similar histology in the xenograft and primary tumors. This finding indicates that CD133^+^ cells exhibit powerful tumorigenicity *in vitro* (Fig. 6C).

## Discussion

According to the CSC theory, lung cancer may be described as a stem cell disease originating from the malignant transformation of adult lung stem cells; such transformed adult stem cells are also known as LCSCs. To date, three types of LCSC have been reported. Ho *et al* (18) reported that LCSCs expressing ABC membrane transporter proteins such as ABCG2, multidrug resistance-associated protein 1 and multidrug resistance protein 1 were predominantly localized in SP cells, while Eramo *et al* (19) reported that LCSCs are a subset of CD133^+^ cells. By contrast, Dong *et al* (21) identified that BASC-like LCSCs were present in human pulmonary adenocarcinoma samples. These cells were characterized by a CD24^+^/IGF^−^IR^+^ phenotype and expressed a variety of genes that comprise the backbone of embryonic and lung stem cells; consequently, these cells were highly invasive and tumorigenic.

Research into LCSCs is in the preliminary stages and LCSCs are not yet commercialized; therefore, they are difficult to purchase and studies of LCSCs generally require an initial isolation step. There are currently four methods for the separation or enrichment of CSCs: i) Separation of SP cells (18); ii) separation using flow cytometry or magnetic beads with recognized cell surface markers (19); iii) suspension of the culture in serum-free medium; and iv) selection based on the drug resistance of LCSCs, which increases the purity of stem cells by promoting the apoptosis of other SP cells through *in vitro* or *in vivo* treatment with therapeutic agents (22). However, these four separation methods have certain limitations. For example, Hoechst 33342 is used to separate SP cells based on the characteristics of CSCs, resulting in cellular toxicity, thus, restricting its further application. In addition, no recognized cell surface markers of LCSCs are currently available, therefore, the reliability of cell surface marker separation using flow cytometry or magnetic beads is yet to be confirmed. Furthermore, the sorting process may cause damage to the cells and the number of sorted cells may be small due to a low proportion of positive cells in the sorted tissues. Moreover, suspension of the culture in serum-free medium alone may only achieve preliminary enrichment of CSCs. Thus, other SP cells may be present, resulting in a culture with reduced LCSC purity and concentration that is unsuitable for further research. Additionally, cell cultures are time-consuming and costly. Finally, the CSC theory is in the exploratory stage; the process by which LCSCs initiate tumorigenesis, as well as the molecular mechanisms of their formation and existence, particularly CSC self-regulation mechanisms, have yet to be investigated. Hence, the induction of resistant cells may affect the biological properties of CSCs (23–25).

Considering that the undifferentiated LCSCs are more resistant to apoptosis and more able to withstand serum-free conditions than human lung cancer cells, the present experiment initially used flow cytometry to detect the proportion of CD133^+^ cells in single cell suspensions of sorted lung cancer tissue samples. Subsequently, the single cell suspensions containing the highest proportion of CD133^+^ cells were selected and the CD133^+^ cells were isolated using immunomagnetic beads. Utilizing the characteristics of LCSCs, such as resistance to serum-free culture conditions and apoptosis tolerance, a serum-free suspension culture was used to enrich the CD133^+^ cells in the population. Following greater than one week of culturing, the cells were observed to form a ball. The two techniques proved complementary, producing a novel method that should be further investigated, due to the short culture period, low cost and high efficiency.

The functions of the cultured cells were predominantly used to determine whether they possessed the characteristics of LCSCs. The present study identified that, following enrichment, the CD133^+^ tumor spheres exhibited strong self-renewal capacities compared with the CD133^−^ cells. In the serum-free culture, 82.3% of CD133^+^ cells formed balls of stem cells, which was significantly different from the CD133^−^ cells (1.21%; P<0.01). In addition, the CD133^+^ cells possessed multipotent differentiation capacity. The expression patterns of CK8 and CK18 following cell differentiation were similar to those observed in the primary cell culture. CK8 and CK18 are important protein components of the cytoskeleton, and are the most widely expressed members of the 21 intermediate filament types of epithelial and tumor cells (26). CK8 and CK18 function in the determination and maintenance of cell morphology, and the regulation of spatial organization of proteins in cell organelles and the cytoplasm. Furthermore, CK8 and CK18 participate in cell movement, cell division and cytoplasmic transport (27). Positive expression of CK8 and CK18 is a diagnostic indicator of pulmonary adenocarcinoma in the pathological diagnosis of lung cancer (28). The results of the present study demonstrated that chemotherapeutic agent resistance was significantly higher in CD133^+^ cells compared with in CD133^−^ cells and, thus, is a characteristic of CSCs (29).

The gold standard for determining whether LCSCs are present in an enriched cell population is the tumorigenicity experiment in NOD/SCID mice (19). The present study used this method to demonstrate that CD133^+^ cells exhibit higher tumorigenic capacities than CD133^−^ cells. The transplantation of only 100 CD133^+^ cells was sufficient to form tumors under the abdominal wall of NOD/SCID mice. When the same numbers of CD133^+^ and CD133^−^ cells were transplanted under the abdominal wall of NOD/SCID mice, the tumor volumes formed by CD133^+^ cells were significantly greater than those formed by CD133^−^ cells.

In conclusion, the culture and identification of LCSCs remains a controversial topic. To the best of our knowledge, the present study is the first to use flow cytometry to detect the proportion of CD133^+^ cells in single cell suspensions of lung cancer tissue and to use immunomagnetic beads to select the single cell suspensions with the highest proportions of CD133^+^ cells. Used in combination with serum-free suspension culture, this technique enriched the LCSC population sufficiently to overcome the limitations of the existing separation CSC separation techniques: The existence of other SP cells; the time-consuming characteristic of serum-free suspension culture; and the difficulties of immunomagnetic separation due to a low proportion of positive cells in the sorted cell population. The present method improves the speed of LCSC separation, resulting in increased purity and concentration of LCSC. Furthermore, the self-renewal, multipotent differentiation capacity, drug resistance and tumorigenic capacity of LCSCs were verified experimentally, providing a foundation for further theoretical research into LCSCs.

## Figures and Tables

**Table I:** Case description and CD133 expression in eight NSCLC patients.

Patient	Age, years	Gender	Tumor subtype	TNM stage	CD133 proportion, %
1	73	Male	SCC	IIA	None detected
2	56	Female	AdC	IIIA	1.9
3	65	Female	AdC	IIA	None detected
4	61	Male	AdC	IIB	2.1
5	57	Male	AdC	IIB	None detected
6	74	Male	AdC	IIIA	1.3
7	69	Male	SCC	IIB	None detected
8	58	Male	AdC	IIA	0.8

TNM staging according to International Association for the Study of Lung Cancer Lung Cancer Staging Project (20). CD, cluster of differentiation; NSCLC, non-small cell lung cancer; TNM, tumor node metastasis; SCC, squamous-cell carcinoma; AdC, adenocarcinoma.

**Table II:** Incidence of tumors in non-obese diabetic/severe combined immunodeficiency mice serially transplanted with CD133^+^ and CD133^−^ cells.

	Cells, n			
	
Transplanted cells	10^2^	10^3^	10^4^	10^5^
CD133^−^	0/5	0/5	2/5	4/5
CD133^+^	2/5	4/5	5/5	5/5

CD, cluster of differentiation.
